# Predicting Thromboembolism in Hospitalized Patients with Ventricular Thrombus

**DOI:** 10.31083/j.rcm2312390

**Published:** 2022-11-30

**Authors:** Qing Yang, Xin Quan, Xinyue Lang, Yan Liang

**Affiliations:** ^1^National Clinical Research Center of Cardiovascular Diseases, Fuwai Hospital, National Center for Cardiovascular Diseases, Chinese Academy of Medical Sciences and Peking Union Medical College, 100037 Beijing, China; ^2^Emergency Center, Fuwai Hospital, National Center for Cardiovascular Diseases, Chinese Academy of Medical Sciences and Peking Union Medical College, 100037 Beijing, China; ^3^Echocardiographic Imaging Center, Fuwai Hospital, National Center for Cardiovascular Diseases, Chinese Academy of Medical Sciences and Peking Union Medical College, 100037 Beijing, China; ^4^Medical Research & Biometrics Center, National Center for Cardiovascular Diseases, Chinese Academy of Medical Sciences, 102300 Beijing, China

**Keywords:** ventricular thrombus, prediction model, thromboembolism

## Abstract

**Background:**

Thromboembolism is associated with mortality and morbidity in patients with ventricular thrombus. Early detection of thromboembolism is critical. This study aimed to identify potential predictors of patient characteristics and develop a prediction model that predicted the risk of thromboembolism in hospitalized patients with ventricular thrombus.

**Methods:**

We performed a retrospective cohort study from the National Center of Cardiovascular Diseases of China between November 2019 and December 2021. Hospitalized patients with an initial diagnosis of ventricular thrombus were included. The primary outcome was the rate of thromboembolism during the hospitalization. The Lasso regression algorithm was performed to select independent predictors and the multivariate logistic regression was further verified. The calibration curve was derived and a nomogram risk prediction model was built to predict the occurrence of thromboembolism.

**Results:**

A total of 338 eligible patients were included in this study, which was randomly split into a training set (n = 238) and a validation set (n = 100). By performing Lasso regression and multivariate logistic regression, the prediction model was established including seven factors and the area under the receiving operating characteristic was 0.930 in the training set and 0.839 in the validation set. Factors associated with a high risk of thromboembolism were protuberant thrombus (odds ratio (OR) 5.03, 95% confidential intervals (CI) 1.14–23.83, *p* = 0.033), and history of diabetes mellitus (OR 6.28, 95% CI 1.59–29.96, *p* = 0.012), while a high level of left ventricular ejection fraction along with no antiplatelet therapy indicated a low risk of thromboembolism (OR 0.95, 95% CI 0.89–1.01, *p* = 0.098; OR 0.26, 95% CI 0.05–1.07, *p* = 0.083, separately).

**Conclusions:**

A prediction model was established by selecting seven factors based on the Lasso algorithm, which gave hints about how to forecast the probability of thromboembolism in hospitalized ventricular thrombus patients. For the development and validation of models, more prospective clinical studies are required.

**Clinical Trial Registration:**

NCT 05006677.

## 1. Introduction

It has long been a topic of discussion in medical settings on how to prevent thromboembolism, particularly cardiac embolism. Researchers reported that patients with ventricular thrombus had a high risk of stroke or systemic embolism (SSE) more than 20% before being discharged despite anticoagulation [1, 2, 3], and studies indicated that the in-hospital mortality rate of patients with ventricular thrombus was higher compared to patients without ventricular thrombus [4, 5]. With the advanced technology in imaging tools, the incidence of ventricular thrombus has increased in recent years, with a range of 4%–10% [6, 7]. As thromboembolism is currently the most noteworthy severe outcome in patients with ventricular thrombus [8, 9], it is of vital importance to identify which patients are at a higher risk of thromboembolism, tending to decrease mortality or mobility. Prediction models in the prevention of atrial fibrillation (AF)-related stroke have been developed [10, 11, 12], up to date, there is no prediction model built on the theme of thromboembolism secondary to ventricular thrombus, especially focusing on hospitalized medical patients. In our study, we aimed to build a prediction model by analyzing potential predictors including clinical characteristics, laboratory data, or imaging measurements, to better help clinicians target early awareness in hospitalized patients with high-risk factors, as well as to provide provoking thoughts or evidence in the management of patients with ventricular thrombus.

## 2. Methods

### 2.1 Patient Population

This retrospective cohort study was conducted from November 2019 to December 2021 using electronic medical records of Fuwai Hospital, National Center of Cardiovascular Diseases in China, which was registered in ClinicalTrials.gov: NCT 05006677. This prediction model study was reported in accordance with the TRIPOD checklist [13]. The inclusion criteria were: (1) Age ≥18 years; (2) Patients admitted to the center with the initial diagnosis of ventricular thrombus or occurred ventricular thrombus during the hospitalization. Patients diagnosed with inherited or acquired thrombophilia (e.g., antiphospholipid syndrome) were excluded since the risk of thromboembolism in these patients was established on a unique pathophysiological mechanism.

### 2.2 Definitions

The diagnosis of ventricular thrombus was confirmed by transesophageal or transthoracic echocardiography with or without contrast, computer tomography (CT), or cardiac magnetic resonance (CMR) imaging. When these imaging tools were not consistent, X.Q. (Ph.D., majoring in echocardiography) and other professors would review images and reach a conclusion. A ventricular thrombus was identified as a ventricular cavity with an aberrant echo mass or intensity, whose edge was different from the ventricular endocardium [14]. The existence of the thrombus was confirmed by several sections, including parasternal short and long-axis views, as well as apical 2-, 3-, and 4-chamber images. When a thrombus was detected, its morphology was categorized as either mural (if its borders are generally continuous with the adjacent endocardium) or protuberant (if its borders are distinct from the adjacent endocardium and protrude into the ventricular cavity) [15].

Information on thromboembolism events during the hospitalization was obtained by searching our institutional database. Thromboembolism events were defined as the composite of ischemic stroke or transient ischemic attack, pulmonary embolism (PE), and systemic embolic events, with the exclusion of deep venous thrombosis [16]. Ischemic stroke and transient ischemic attack were defined as the presence of acute focal neurological deficit with clinical symptoms or signs [17]. PE and peripheral embolic events were documented by angiography or objective testing [18].

### 2.3 Model Development

Two colleagues (Q.Y. and X.Q.) extracted the data independently and compared the results to ensure coherence, and an additional scholar resolved the discrepancies. A total of 46 variables including patient demographics, laboratory results, and imaging measurements were collected in the initial model.

The data were randomly split into a training set (70% of the sample) and a validation set (30% of the sample). The training set was the terminology used in univariate regression as well as Lasso regression to find out clinical potential factors. Variables with a *p* value < 0.10 in univariate analysis were considered to be linked to the outcome and then performed stepwise predictor selection in three directions separately (forward, backward, and both), defined as Model 1 followed by multiple logistic regression. Odds ratio (OR) and 95% confidence interval (CI) were calculated using logistic regression models. We also conducted Lasso regression with L1-penalized least absolute shrinkage to select other potential factors and then formed Model 2 by performing multivariate analysis based on the Lasso method. The reliability of the predictive model was assessed concerning discrimination and calibration. The discrimination analysis and the mean area under the receiver operating characteristic curve (AUROC) obtained by repeated cross-validation (ten-fold), were used to select models. This procedure was repeated many times and the performance on the validation set was averaged to select the model with the greatest external validity. The reliability of the model was then evaluated using a concordance index (C-index) and a calibration plot via the bootstrap method which was tested with a Hosmer-Lemeshow goodness-of-fit test ($R^{2}$) [19]. The regression model with the minimum Akaike’s information criterion was used in the nomogram formulation. To quantitatively visualize the net benefit of clinical decisions, the decision curve analysis (DCA) was also conducted.

### 2.4 Statistics Analysis

Descriptive statistics were computed using the CBCgrps-Package in R [20]. Continuous variables were presented as mean (standard deviation, SD) or median (interquartile range, IQR) and as frequency (percentage) for categorical variables [21]. Analysis of variance was used to compare normally continuous variables and Pearson chi-squared test for categorical data. The Fisher exact test and Kruskal-Wallis H test were used as appropriate. Missing data for predictor variables were handled by using multiple imputations by chained equations with predictive mean matching (MICE-Package in R) creating 5 imputed data sets. Categorical variables were encoded by binary with the first category dropped. The car package in R was used to detect collinearity between variables, and a variance inflation factor <10 was tolerated. All analyses were scheduled for completion with R Studio and R, Version 3.5.1 (The R Project for Statistical Computing, Vienna, Austria).

## 3. Results

### 3.1 Patients Characteristics

A total of 498 patients were identified in the electronic records from November 2019 to December 2021, while 7 out of 498 patients were without ventricular thrombus. 153 patients were excluded, of these, 136 patients were already diagnosed with ventricular thrombus before this hospitalization, 12 patients were aged <18 years, and 5 patients had a suspected diagnosis of thrombophilia (2 antiphospholipid syndrome) at discharge. Overall, we included 338 eligible patients in this study, which were randomly split into a training set (n = 238) and a validation set (n = 100) (**Supplementary Fig. 1**). Among 338 patients, 20 (5.9%) patients underwent thrombectomy therapy, either with or without ventricular aneurysm resection, and 9 (2.7%) patients had heart transplantation in the hospital. 288 (85.2%) patients were male and 71 (21%) patients were overweight (defined as body mass index (BMI) ≥28%). Patients who were diagnosed with myocardial infarction (MI) at admission accounted for 62% (n = 208). At baseline, the median level of D-dimer was more than two-fold higher than the reference value (<0.5 g/L) while the level of fibrin degradation products (FDP) with a median range of 2.6 (IQR 2.5, 5.5) g/L was negatively normal (0–5 g/L). Most patients (79.9%) had a creatinine clearance (CrCl) of more than 50 mL/min while 54 (16%) patients had moderate renal dysfunction with the range of 30 mL/min to 49 mL/min and 14 (4.1%) patients had a CrCl of less than 30 mL/min. The median of N-Terminal pro-brain natriuretic peptide (NT-proBNP) was 2408.0 pg/mL, and 123 (36.4%) patients had a more than 10% decline in NT-proBNP at discharge (**Supplementary Table 1**).

In our study, 282 (83.4%) patients were diagnosed with ventricular thrombus confirmed by echocardiography and 13 (6.8%) patients depended on CMR to find ventricular thrombus while their echocardiograms were negative. Another 43 (12.7%) patients had a record of ventricular thrombus only with CT in our center. Patients had a median left ventricular ejection fraction (LVEF) of 35.0% and a left ventricular end-diastolic diameter of 60 mm. 287 (85%) patients had a mural thrombus, and the remaining patients had a protuberant thrombus with or without a mobile free edge. In terms of anticoagulation therapy, 176 patients (52%) had heparin injections whereas 239 patients (71%) received oral anticoagulation during the period of hospitalization, of which 72% were on non-vitamin K antagonist oral anticoagulants (NOACs) and 28% on warfarin. Of the 173 patients who took NOACs, 165 (95.4%) received rivaroxaban (almost half of whom took 20 mg daily), and the remaining 8 (4.6%) were given dabigatran 110 mg twice daily. Given the high percentage of patients with coronary artery diseases, 164 (49%) patients got antiplatelet therapy, with 86 receiving mono antiplatelet therapy (20 on aspirin and 76 on clopidogrel) and 78 receiving dual antiplatelet therapy (66 on aspirin plus clopidogrel and 12 on aspirin plus ticagrelor). Above all, no significant differences were found comparing the training cohort and validation cohort in demography and clinic characteristics (Table 1).

**Table 1.** **Clinical characteristics of patients with ventricular thrombus in the training group and validation group**.

			Total (N = 338)	Training group (N = 238)	Validation group (N = 100)	*p* value
Age, y			54.6 ± 14.7	54.8 ± 14.6	54.2 ± 15.2	0.753
Male, n (%)			288 (85.2)	205 (86.1)	83 (83)	0.567
Weight, kg			72.4 ± 14.3	71.5 ± 13.7	74.4 ± 15.4	0.111
BMI, kg/$m^{2}$			24.9 ± 4.0	24.7 ± 3.8	25.5 ± 4.4	0.102
Systolic blood pressure, mmHg			117 ± 19	116.1 ± 19.3	119.4 ± 19.6	0.159
Diastolic blood pressure, mmHg			76 ± 11	75.7 ± 11.4	77.1 ± 13.1	0.355
Heart rate, bpm			78 ± 15	77.9 ± 15.2	79.4 ± 17.1	0.471
Length of hospital stay, d			11 (6, 16)	11 (7, 16)	10.5 (5, 16)	0.413
Present diagnosis of MI, n (%)			208 (62)	145 (61)	63 (63)	0.814
Medical history, n (%)						
	Coronary artery disease		242 (72)	168 (71)	74 (74)	0.615
	Atrial fibrillation		35 (10)	27 (11)	8 (8)	0.468
	Heart failure		192 (57)	134 (56)	58 (58)	0.867
	Hypertension		161 (48)	111 (47)	50 (50)	0.656
	Diabetes mellitus		114 (34)	82 (34)	32 (32)	0.757
	Chronic kidney disease		21 (6)	16 (7)	5 (5)	0.725
	SSE		35 (10)	24 (10)	11 (11)	0.955
Laboratory test						
	D-dimer, ug/mL		1.09 (0.42, 2.65)	1.15 (0.49, 2.65)	0.99 (0.36, 2.49)	0.357
	FDP, ug/mL		2.6 (2.5, 5.4)	2.8 (2.5, 5.6)	2.5 (2.5, 4.8)	0.367
	Neutrophil count, ×${\mathrm{10}}^{9}$/L		4.8 (3.7, 6.2)	4.9 (3.7, 6.3)	4.7 (3.8, 6.0)	0.747
	Lymphocyte count, ×${\mathrm{10}}^{9}$/L		1.7 (1.3, 2.2)	1.7 (1.3, 2.2)	1.7 (1.2, 2.3)	0.623
	Platelet count, ×${\mathrm{10}}^{9}$/L		211 (172, 262)	209 (172, 257)	215 (175, 278)	0.589
	C-reactive protein, mg/L		6.1 (2.8, 19.9)	6.1 (2.7, 20.1)	6.2 (2.9, 14.2)	0.645
	APTT, S		38.1 (34.5, 43.1)	38.3 (34.9, 43.0)	37.9 (33.9, 43.2)	0.457
	FIB, g/L		3.6 (3.0, 4.4)	3.6 (3.0, 4.3)	3.6 (3.0, 4.4)	0.979
	PT, S		14.0 (13.1, 16.0)	14.2 (13.2, 16.0)	13.7 (13.0, 15.4)	0.092
	TT, S		16.3 (15.5, 17.8)	16.2 (15.5, 17.8)	16.3 (15.9, 17.7)	0.105
	INR, R		1.08 (0.99, 1.28)	1.10 (1.01, 1.29)	1.06 (0.98, 1.23)	0.100
	PTA, %		87 (68, 101)	86 (68, 99)	91 (72, 103)	0.104
	CrCl, mL/min		66.2 (52.5, 84.1)	65.2 (51.4, 84.3)	66.7 (53.9, 83.1)	0.704
	NT-proBNP, pg/mL		2408.0 (709.1, 7127.0)	2408.0 (682.5, 7205.5)	2437.5 (743.2, 7049.1)	0.959
Imaging measurements						
	LVEF, %		35.0 (26.0, 45.0)	35.5 (26.0, 44.7)	32.5 (26.0, 45.0)	0.626
	Left ventricular end-diastolic diameter, mm		60 (53, 68)	60 (53, 68)	60 (54, 70)	0.484
	Site of thrombus, n (%)					1.000
		Left ventricle	313 (93)	220 (92)	93 (93)	
		Right ventricle	15 (4)	11 (5)	4 (4)	
		Biventricular	10 (3)	7 (3)	3 (3)	
	Amount of thrombus, n (%)					0.307
		1	213 (63)	154 (65)	59 (59)	
		≥2	76 (22)	54 (23)	22 (22)	
		Unknown	49 (14)	30 (13)	19 (19)	
	Thrombus morphology, n (%)					0.389
		Mural	287 (85)	199 (84)	88 (88)	
		Protuberant	51 (15)	39 (16)	12 (12)	
	Spontaneous echo contrast, n (%)		9 (3)	3 (1)	6 (6)	0.022
	Regional wall motion abnormality, n (%)		182 (54)	126 (53)	56 (56)	0.693
	Ventricular aneurysm, n (%)		161 (48)	115 (48)	46 (46)	0.787
	Echo intensity, n (%)					0.074
		Low	47 (21)	40 (24)	7 (12)	
		Moderate	109 (49)	74 (45)	35 (58)	
		High	67 (30)	49 (31)	18 (30)	
	Revascularization, n (%)		71 (21)	47 (20)	24 (24)	0.466
	Antiplatelet therapy, n (%)		164 (49)	111 (47)	53 (53)	0.343
	Heparin, n (%)		176 (52)	129 (54)	47 (47)	0.276
	Anticoagulation therapy, n (%)					0.876
		None	99 (29)	70 (29)	29 (29)	
		NOACs	173 (51)	120 (50)	53 (53)	
		Warfarin	66 (20)	48 (20)	18 (18)	

Variables are presented as n (%), mean ± SD, and median (IQR). Abbreviations: N, numbers of patients; SD, standard deviation; IQR, interquartile range; BMI, body mass index; MI, myocardial infarction; SSE, stroke or systemic embolism; FDP, fibrin degradation products; APTT, activated partial thromboplastin time; PT, prothrombin time; TT, thrombin time; INR, international normalized ratio; FIB, fibrinogen; PTA, prothrombin activity; CrCl, creatinine clearance; NT-proBNP, N-Terminal pro-brain natriuretic peptide; LVEF, left ventricular ejection fraction; NOACs, non-vitamin K antagonist oral anticoagulants.

### 3.2 Factors Selected by Univariate and Lasso Regression

**Fig. 1.**
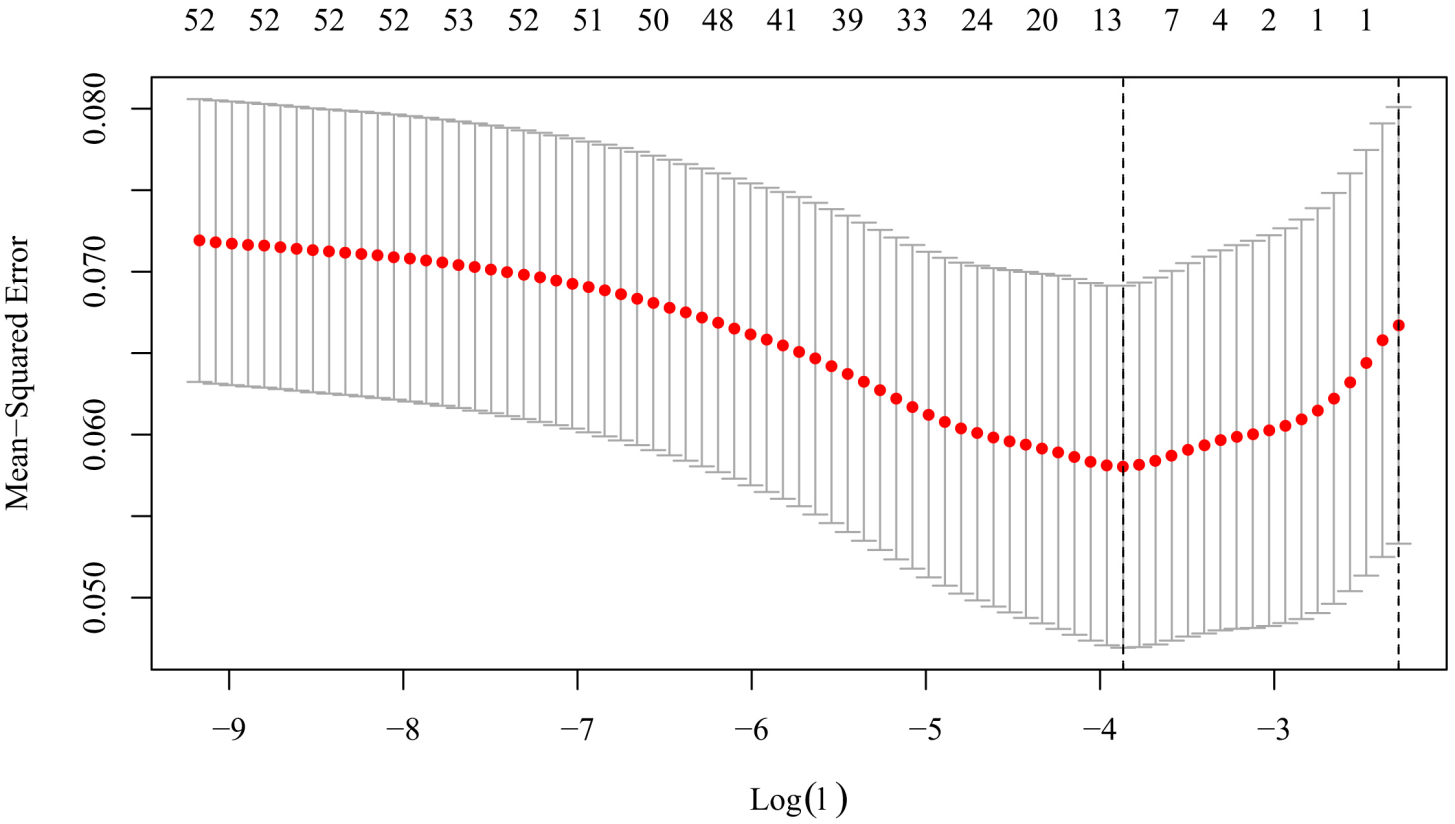
**Tuning parameter (Lambda) selection in the Lasso Model used ten-fold cross-validation based on the minimum criteria (left dotted vertical line) or the 1 standard error criteria (right dotted vertical line)**.

**Fig. 2.**
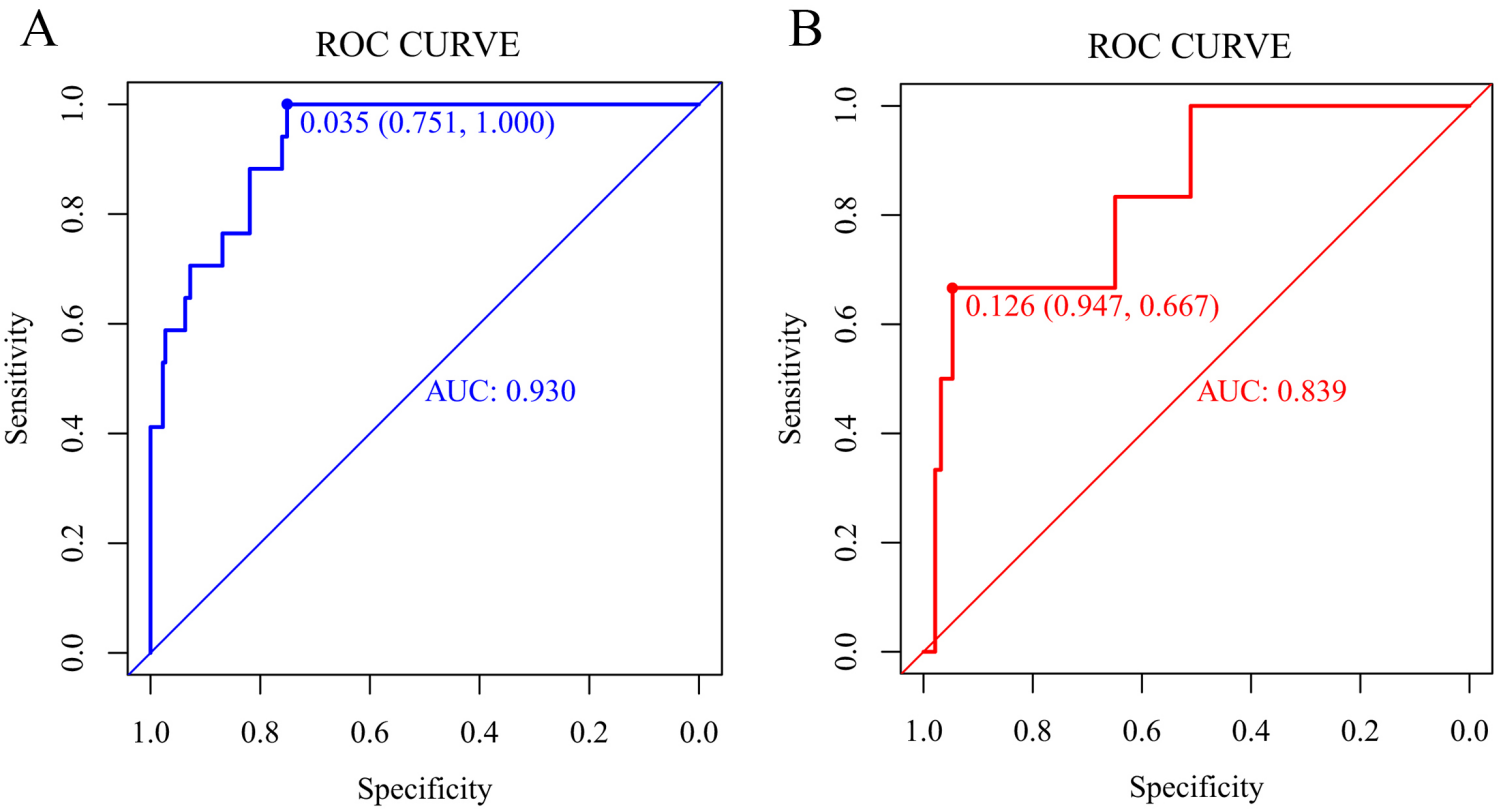
**ROC curvesof Model 2 for predicting the risk of thromboembolism**. (A) Training set. (B) Validation set. ROC, receiver operating characteristic; AUC, area under the ROC curve.

We included 46 characteristics in our models. A total of 15 factors were selected from the univariate analysis (Table 2) and 5 factors remained after performing a multiple logistic regression model which formed Model 1 (Table 3). They were BMI, ventricular aneurysm, history of diabetes mellitus (DM), prior SSE, and therapy of antiplatelet. And with the Lasso regression, Lambda = 0.000010 was chosen (minimum criteria) according to ten-fold cross-validation of the Lasso coefficient profiles of the 46 features, and 11 factors were selected (Fig. 1 and **Supplementary Fig. 2**). A multiple logistic regression model was established using Lasso regression and the analysis results were shown in Table 3. The following four risk factors were not associated with the outcome (*p *< 0.05): history of heart failure (HF), therapy of heparin, site of thrombus, and FDP change. Finally, a total of 7 factors (BMI, diastolic blood pressure, LVEF, thrombus morphology, medical history of DM, prior SSE, and antiplatelet therapy) were extracted into Model 2. By comparing the AUROC, Model 2 showed a greater AUROC in the training set than Model 1 (Model 1: 0.904, 95% CI 0.850–0.958; Model 2: 0.930, 95% CI 0.883–0.977, *p* = 0.205), as well as Model 2 performed better in the validation set (Model 1: 0.805, 95% CI 0.609–1.000; Model 2: 0.839, 95% CI 0.669–1.000, *p* = 0.354) (Fig. 2, and **Supplementary Figs. 3,4**). Positive agreements between ideal curves and calibration curves were also observed **Supplementary Figs. 5,6**). The DCA curve revealed a range of cutoff probabilities shown by the nomogram (**Supplementary Fig. 7**). In summary, we chose Model 2 as the final model to make a prediction. The prediction result of Model 2 after incorporating the 7 factors into the model was presented in Fig. 2 with the AUROC being 0.930 in the training set and 0.839 in the validation set in Model 2. And by conducting the leave-one-out cross-validation, the accuracy of Model 2 was 0.937 while the Kappa value was 0.413.

**Table 2.** **Characteristics of patients with or without thromboembolism events in hospital and the univariate logistic regression analysis**.

Variable			No event (N = 221)	Event (N = 17)	Univariable	
					OR ${\mathrm{(95\% CI)}}^{†}$	*p* value
Age			54.9 ± 14.6	53.6 ± 15.2	0.99 (0.96–1.03)	0.765
Male (vs female)			190 (86)	15 (88.2)	0.82 (0.18–3.75)	0.795
Weight			72.0 ± 13.8	65.6 ± 11.2	0.97 (0.93–1.00)	0.064
BMI			24.8 ± 3.9	22.5 ± 2.8	0.85 (0.74–0.97)	0.016
Systolic blood pressure			116 ± 19	112 ± 17	0.99 (0.96–1.02)	0.407
Diastolic blood pressure			75 ± 11	80 ± 18	1.03 (0.99–1.07)	0.133
Heart rate			77 ± 15	84 ± 19	1.03 (0.99–1.06)	0.107
Present diagnosis of MI			136 (61.5)	9 (52.9)	0.70 (0.26–1.89)	0.486
Length of hospital stay			12 (7, 16)	10 (8, 17)	1.01 (0.96–1.06)	0.769
Medical history						
	Coronary artery disease		157 (71)	11 (64.7)	0.75 (0.26–2.11)	0.582
	Atrial fibrillation		27 (12.2)	0 (0)	NA	0.990
	Heart failure		119 (53.8)	15 (88.2)	6.43 (1.44–28.79)	0.015
	Hypertension		103 (46.6)	8 (47.1)	1.02 (0.38–2.73)	0.971
	Diabetes mellitus		72 (32.6)	10 (58.8)	2.96 (1.08–8.08)	0.035
	Chronic kidney disease		15 (6.8)	1 (5.9)	0.86 (0.11–6.92)	0.886
	SSE		15 (6.8)	9 (52.9)	15.45 (5.21–45.85)	<0.001
Laboratory test						
	D-dimer		1.04 (0.47, 2.51)	2.75 (1.14, 4.34)	1.12 (1.00–1.26)	0.041
	D-dimer at discharge					
		-1 ∼ +1fold	133 (60.2)	10 (58.8)	Reference	
		+1fold∼	29 (13.1)	0 (0)	NA	0.989
		∼-1fold	59 (26.7)	7 (41.2)	1.58 (0.57–4.35)	0.378
	FDP		2.7 (2.5, 5.2)	6.3 (2.5, 10.3)	1.01 (0.99–1.04)	0.345
	FDP change					
		-1 ∼ +1fold	179 (81)	12 (70.6)	Reference	
		+1fold∼	20 (9)	1 (5.9)	0.75 (0.09–6.04)	0.783
		∼-1fold	22 (10)	4 (23.5)	2.71 (0.80–9.14)	0.108
	Neutrophil count		4.8 (3.6, 6.1)	5.8 (4.9, 6.6)	1.16 (0.95–1.43)	0.143
	Lymphocyte count		1.7 (1.3, 2.2)	1.4 (1.0, 2.0)	0.41 (0.17–0.97)	0.043
	Platelet count		214 (171, 259)	185 (179, 218)	1.00 (0.99–1.00)	0.282
	C-reactive protein, mg/L		5.9 (2.7, 19.5)	17.6 (6.1, 38.5)	1.00 (1.00–1.01)	0.491
	APTT, S		38.2 (34.9, 43.1)	38.8 (36.6, 40.3)	0.97 (0.89–1.04)	0.372
	FIB, g/L		3.6 (3.0, 4.3)	3.6 (2.9, 4.4)	1.10 (0.74–1.62)	0.646
	PT, S		14.2 (13.2, 15.8)	14.5 (13.8, 16.7)	1.03 (0.91–1.16)	0.629
	TT, S		16.2 (15.5, 17.8)	16.4 (15.5, 18.6)	0.98 (0.88–1.08)	0.635
	INR, R		1.09 (1.00, 1.27)	1.14 (1.07, 1.34)	1.29 (0.43–3.88)	0.656
	PTA, %		87 (68, 99)	81 (63, 89)	0.99 (0.97–1.01)	0.279
	CrCl, mL/min		66.0 (52.0, 84.3)	61.4 (50.2, 78.1)	1.00 (0.98–1.01)	0.690
	NT-proBNP		2292.0 (600.0, 6387.0)	8051.0 (2596.0, 11742.9)	1.00 (0.99–1.00)	0.053
	NT-proBNP at discharge (Ref baseline)					
		-1 ∼ +1fold	112 (50.7)	10 (58.8)	Reference	
		+1fold∼	32 (14.5)	0 (0)	NA	0.989
		∼-1fold	77 (34.8)	7 (41.2)	1.02 (0.37–2.79)	0.972
Imaging measurements						
	LVEF, %		36 (28, 45)	26 (20, 34)	0.94 (0.89–0.98)	0.010
	Left ventricular end-diastolic diameter, mm		59 (53, 67)	63 (58, 75)	1.04 (1.00–1.08)	0.060
	Site of thrombus					
		Left ventricle	207 (93.7)	13 (76.5)	Reference	
		Right ventricle	10 (4.5)	1 (5.9)	1.59 (0.19–13.41)	0.669
		Biventricular	4 (1.8)	3 (17.6)	11.94 (2.41–59.11)	0.002
	Amount of thrombus					
		1	146 (66.1)	8 (47.1)	Reference	
		≥2	47 (21.3)	7 (41.2)	2.72 (0.94–7.89)	0.066
	Thrombus morphology					
		Mural	187 (84.6)	12 (70.6)	Reference	
		Protuberant	34 (15.4)	5 (29.4)	2.29 (0.76–6.92)	0.141
	Spontaneous echo contrast		3 (1.4)	0 (0)	NA	0.992
	Regional wall motion abnormality, n (%)		121 (54.8)	5 (29.4)	0.34 (0.12–1.01)	0.052
	Ventricular aneurysm, n (%)		112 (50.7)	3 (17.6)	0.21 (0.06–0.75)	0.016
	Echo intensity					
		Low	37 (16.7)	3 (17.6)	Reference	
		Moderate	69 (31.2)	5 (29.4)	0.89 (0.20–3.95)	0.882
		High	45 (20.4)	4 (23.5)	1.10 (0.23–5.21)	0.908
	Revascularization, n (%)		47 (21.3)	0 (0)	NA	0.991
	Antiplatelet therapy, n (%)		108 (48.9)	3 (17.6)	0.22 (0.06–0.80)	0.021
	Heparin, n (%)		123 (55.7)	6 (35.3)	0.43 (0.16–1.22)	0.113
	Anticoagulation therapy					
		None	66 (29.9)	4 (23.5)	Reference	
		NOACs	109 (49.3)	11 (64.7)	1.67 (0.51–5.44)	0.399
		Warfarin	46 (20.8)	2 (11.8)	0.72 (0.13–4.08)	0.708

Variables are presented as n (%), mean ± SD, and median (IQR). ^†^NA was presented when the sample was zero in comparison groups. Abbreviations: N, numbers of patients; SD, standard deviation; IQR, interquartile range; OR, odds ratio; CI, confidence interval; BMI, body mass index; MI, myocardial infarction; SSE, stroke or systemic embolism; FDP, fibrin degradation products; APTT, activated partial thromboplastin time; PT, prothrombin time; TT, thrombin time; INR, international normalized ratio; FIB, fibrinogen; PTA, prothrombin activity; CrCl, creatinine clearance; NT-proBNP, N-Terminal pro-brain natriuretic peptide; LVEF, left ventricular ejection fraction; NOACs, non-vitamin K antagonist oral anticoagulants.

**Table 3.** **Two models based on multivariate logistic analysis with univariate analysis (Model 1) or Lasso regression (Model 2)**.

Variable	Model 1		Model 2	
	OR (95% CI)	*p* value	OR (95% CI)	*p* value
BMI	0.80 (0.66–0.95)	0.017	0.76 (0.59–0.94)	0.018
Diastolic blood pressure	–	–	1.07 (1.01–1.14)	0.019
LVEF	–	–	0.95 (0.89–1.01)	0.098
Thrombus morphology				
Protuberant vs mural	–	–	5.03 (1.14–23.83)	0.033
Ventricular aneurysm	0.33 (0.06–1.32)	0.141	–	–
Prior SSE	15.23 (4.39–59.46)	<0.001	53.78 (10.76–394.56)	<0.001
Medical history of DM	5.17 (1.54–19.78)	0.010	6.28 (1.59–29.96)	0.012
Antiplatelet therapy	0.36 (0.07–1.42)	0.174	0.26 (0.05–1.07)	0.083

Abbreviations: OR, odds ratio; CI, confidence interval; BMI, body mass index; LVEF, left ventricular ejection fraction; SSE, stroke or systemic embolism; DM, diabetes mellitus.

### 3.3 Prediction Model in the Prediction of Thromboembolism

**Fig. 3.**
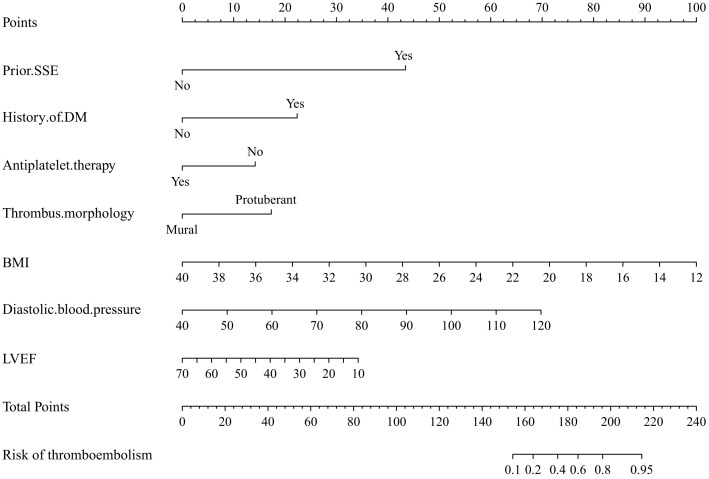
**Nomogram for the prediction of the outcome of thromboembolism in Model 2**. Model 2: Prior SSE + Medical history of DM + Antiplatelet therapy + Thrombus morphology + Diastolic blood pressure + BMI + LVEF. SSE, stroke or systemic embolism; DM, diabetes mellitus; BMI, body mass index; LVEF, left ventricular ejection fraction.

According to Model 2 (factors included *prior SSE, medical history of DM, thrombus morphology, diastolic blood pressure, BMI, LVEF, and antiplatelet therapy*), we established a nomogram risk prediction model containing independent risk factors ($R^{2}$ 0.52, C index 0.93, 95% CI 0.87–0.99) (Fig. 3). The scores of the items displayed in the nomogram should be added up. For example, if a patient with ventricular mural thrombus, had a level of BMI of 28 ${\mathrm{kg/m}}^{2}$ and diastolic blood pressure of 70 mmHg, had no medical history of DM or SSE, had a level of LVEF of 30%, and he/she was not on antiplatelet therapy during the one-week hospitalization, then the total score was approximately 106, indicating an estimated thromboembolism event of <10%. And considering the wide CI in the factors of the prior SSE, the results needed to be critically evaluated, which could be accounted for by the very small sample of patients who had a history of SSE. Other factors that were related to a high risk of thromboembolism were protuberant thrombus (OR 5.03, 95% CI 1.14–23.83, *p* = 0.033), a higher level of diastolic blood pressure (OR 1,07, 95% CI 1.01–1.14, *p* = 0.019), and history of DM (OR 6.28, 95% CI 1.59–29.96, *p* = 0.012), while a relatively high level of BMI or LVEF along with no antiplatelet therapy indicated a low risk of thromboembolism (OR 0.76, 95% CI 0.59–0.94, *p* = 0.018; OR 0.95, 95% CI 0.89–1.01, *p* = 0.098; OR 0.26, 95% CI 0.05–1.07, *p* = 0.083, separately).

## 4. Discussion 

Our study first conducted a prediction model established on Lasso regression to predict the risk of thromboembolism in hospitalized patients with ventricular thrombus. And we concluded that patients were more likely to experience thromboembolism in hospital, who had a medical history of SSE and DM, a lower BMI and LVEF but a higher diastolic blood pressure at baseline, along with protuberant thrombus and without antiplatelet therapy during hospitalization.

It is well established that DM and prior SSE have been widely used to stratify the risk of stroke, which were proved to be predictors of thromboembolism events in the study. Patients with DM had a higher risk of thrombotic events due to the pathophysiological underpinnings of endothelial dysfunction and vascular inflammation. Recurrent thromboembolism was more common among patients who had previously experienced it, and its incidence was seven times greater than that of newly discovered cases. Patients with a first PE had more than a two-fold risk of developing a second PE [22]. In the model built on the ROCKET-AF trial, prior thromboembolism was the strongest independent predictor of thromboembolism [10], which was similar to our results. Along with a history of DM and stroke, we observed a strong relationship between the history of HF and the occurrence of thromboembolism in univariate analysis, whereas HF has been identified as a risk factor for thromboembolic events in previous research [23, 24]. Patients who experienced HF or cardiac dysfunction (e.g., a high NT-proBNP, a low LVEF, or a large left ventricular end-diastolic volume) at baseline, faced a higher rate of thromboembolism, and it could be attributed to complex pathophysiological mechanisms such as neurohormonal activation or decreased myocardial contractility, resulting in an increased vulnerability to thromboses [25]. And the abnormal blood flow as well as other requirements of Virchow’s triad including hypercoagulability, and endothelial injury was satisfied in patients with HF [26, 27]. In a population-based 30-year cohort study, patients with HF had an increased risk of stroke compared with the general population group [28]. And by pooling 2 trials related to HF, researchers reported stroke occurrence in 4.7% of patients with AF and 3.4% of patients without AF [29]. A large prospective study reported that HF hospitalization increased the risk of MI or stroke [30], which provided the clear message that HF should no longer be considered a minor risk factor for thromboembolism.

In summary of studies that predicted the embolism events, factors including the level of D-dimer indicated a higher additional risk besides the major persistent risk factors [22, 23]. D-dimer and FDP levels at admission were significantly related to a high risk of embolism, otherwise, neither D-dimer nor FDP with more than a one-fold increase at discharge had a significant relationship with events in the study. Without a doubt, patients who had a high D-dimer had a higher risk of any embolism events since D-dimer was inherently an indicator of thrombus formation. Interestingly, another laboratory indicator also showed an opposite relationship with thromboembolism. The lower the level of lymphocyte count was, the risk of thromboembolism increased. Whether the level of lymphocyte count could indicate thromboembolism remained unknown, and more evidence or mechanism is needed to explore. It reported that in COVID-19 patients the lymphocyte count (*p* = 0.004) showed a lower value in the patients with PE compared with those without PE [31]. And previous studies have concluded that the increased inflammation increased the risk of thromboembolism as well, which mostly happened to patients who had inspiratory diseases [25, 32]. Moreover, researchers found that in 60 patients who developed left ventricular thrombus in COVID-19, 21.5% and 16.9% of patients had stroke events and PE separately, while 12.3% of patients had peripheral arterial embolism [33].

When assessing the effect of the amount or location of thrombus on the risk of thromboembolism, as most patients were diagnosed by echocardiographic assessments, it remained to explore a more accurate embolism rate in CMR or CT or contrast echo since CMR has been regarded as gold criteria could find small and more ventricular thrombus [9]. And patients who had biventricular thrombus were more likely to occur thromboembolism, and one of the reasons might be accounted that they had severe cardiac dysfunction as well as a complex inner condition at admission. In terms of thrombus morphology, protuberant or mobile thrombi were related to a higher risk of embolism compared with mural thrombi, though data on the subtype of thrombus were limited. Researchers demonstrated that transthoracic echocardiography implemented with pulsed wave tissue doppler imaging could provide a more precise definition of mass mobility over visual assessment, and concluded that a ≥10 cm/s mass peak Va was considered the most significant predictor of embolic risk in hospitalized patients [34]. In the 2022 statement for left ventricular thrombus [15], researchers suggest that for a protuberant thrombus as well as a newly diagnosed mural thrombus, it would be prudent to give anticoagulation therapy. And a shared decision-making approach is recommended for organized or calcified thrombi.

Generally, patients with ventricular thrombus ought to be governed by anticoagulation in the absence of contraindications. More than 70% of patients received oral anticoagulation and nearly 50% were on heparin in hospital. Patients who had no history of AF were less likely to be pretreated with anticoagulants, which increased the risk of thromboembolism without long-term anticoagulation [8]. In a pooled meta-analysis of studies of ventricular thrombus after MI, the use of anticoagulants (either warfarin or heparin) reduced the risk of stroke by 81% [35]. On the other hand, the results of studies that compared the use of NOACs to vitamin K antagonists in the prevention of embolism risk were controversial [36, 37, 38], requiring more randomized clinical trials (RCTs) to provide robust evidence. Antiplatelet therapy and anticoagulation therapy, which have different targets, both have an effect on reducing the risk of thromboembolism [39, 40]. Upon the topic of antiplatelet therapy secondary to anticoagulation treatment in the field of prevention of thromboembolism, studies have demonstrated that antiplatelet therapy was effective for the primary prevention of embolism events [41, 42, 43]. Other large RCTs have demonstrated a significant reduction ranging from 20% to 69% in recurrent thromboembolism with aspirin versus placebo after anticoagulants were discontinued in patients with a history of embolic events [44, 45]. But the treatment of triple antithrombotic therapy which was associated with a higher rate of bleeding remained unknown for patients with ventricular thrombus [46]. Personalized management for the prevention and treatment of ventricular thrombus should be developed to take into account of patient characteristics.

Concerning other predictors in the final model of this study, we outlined the findings as follows. A high risk of thromboembolism was linked to higher diastolic blood pressure. In the RE-LY trial’s subgroup analysis, patients with high diastolic blood pressure (≥90 mmHg) had a high risk of developing SSE [47]. The elevated diastolic blood pressure was found to be significantly associated with an increased risk of stroke in another RCT with 22,672 patients, with a 1.5-fold risk for diastolic blood pressure of 80–89 mmHg and a 4-fold risk for 90 mmHg or more [48]. Likewise, a remarkable correlation was observed between the BMI and the outcome of the study. Previous results from three RCT trials (ARISTOTLE [49], ROCKET-AF [50], and ENGAGE AF-TIMI 48 [51]) showed that a higher BMI was independently related to a decreased risk of SSE. The reason for the apparent protective effect of obesity is unclear, and we hypothesized that patients in the higher BMI categories are offered earlier and more intensive treatments to manage the risk of stroke events.

Several limitations were as followed. First, the validation set was based on the same dataset with a small sample, which restricted the power and the practical utility of our model. Second, limited to patient resources, the result of the study could not greatly expand to a large population. Third, even if ventricular thrombus mobility is a major prognostic determinant of increased thromboembolism [34], this retrospective analysis did not include a detailed assessment of thrombotic mass mobility. Additionally, it was also undetermined whether or when to implement a strategy to prevent embolism, since this study focused on developing a novel prediction model to identify patients who were at high risk of embolism.

## 5. Conclusions

This study conducted a prediction model by selecting seven factors based on the Lasso algorithm, aiming to identify the risk prediction of thromboembolism in hospitalized patients with ventricular thrombus. Patients who had a medical history of SSE and DM, a lower level of BMI and LVEF but a higher diastolic blood pressure at baseline, along with protuberant thrombus and without antiplatelet therapy during hospitalization, were more likely to experience thromboembolism in hospital. More prospective clinical trials are required to develop and validate models, and individualized discussion and shared decision-making are of critical importance in managing patients with ventricular thrombus.

## Data Availability

The data will be shared on reasonable request to the corresponding author.

## Abbreviations

SSE, stroke or systemic embolism; CT, computer tomography; CMR, cardiac magnetic resonance; BMI, body mass index; VTE, venous thromboembolism; PE, pulmonary embolism; AF, atrial fibrillation; HF, heart failure; MI, myocardial infarction; LVEF, left ventricular ejection fraction; FDP, fibrin degradation products; CrCl, creatinine clearance; NT-proBNP, N-Terminal pro-brain natriuretic peptide; SD, standard deviation; IQR, interquartile range; OR, odds ratio; CI, confidence interval; AUROC, area under the receiver operating characteristic curve; DCA, decision curve analysis; NOACs, non-vitamin K antagonist oral anticoagulants.

## Supplementary Material

Supplementary material associated with this article can be found, in the online version, at https://doi.org/10.31083/j.rcm2312390.

## References

[1] Hudec S, Hutyra M, Precek J, Latal J, Nykl R, Spacek M (2020). Acute myocardial infarction, intraventricular thrombus and risk of systemic embolism. *Biomedical Papers of the Medical Faculty of the University Palacky, Olomouc, Czechoslovakia*.

[2] Ram P, Shah M, Sirinvaravong N, Lo KB, Patil S, Patel B (2018). Left ventricular thrombosis in acute anterior myocardial infarction: Evaluation of hospital mortality, thromboembolism, and bleeding. *Clinical Cardiology*.

[3] Vaitkus PT, Barnathan ES (1993). Embolic potential, prevention and management of mural thrombus complicating anterior myocardial infarction: a meta-analysis. *Journal of the American College of Cardiology*.

[4] McCarthy CP, Vaduganathan M, McCarthy KJ, Januzzi JL, Bhatt DL, McEvoy JW (2018). Left Ventricular Thrombus after Acute Myocardial Infarction: Screening, Prevention, and Treatment. *JAMA Cardiology*.

[5] van Dantzig J M, Delemarre B J, Bot H, Visser C A (1996). Left ventricular thrombus in acute myocardial infarction. *European Heart Journal*.

[6] Rehan A, Kanwar M, Rosman H, Ahmed S, Ali A, Gardin J (2006). Incidence of post myocardial infarction left ventricular thrombus formation in the era of primary percutaneous intervention and glycoprotein IIb/IIIa inhibitors. A prospective observational study. *Cardiovascular Ultrasound*.

[7] Gianstefani S, Douiri A, Delithanasis I, Rogers T, Sen A, Kalra S (2014). Incidence and Predictors of Early Left Ventricular Thrombus after ST-Elevation Myocardial Infarction in the Contemporary Era of Primary Percutaneous Coronary Intervention. *The American Journal of Cardiology*.

[8] Maniwa N, Fujino M, Nakai M, Nishimura K, Miyamoto Y, Kataoka Y (2018). Anticoagulation combined with antiplatelet therapy in patients with left ventricular thrombus after first acute myocardial infarction. *European Heart Journal*.

[9] Shacham Y, Leshem-Rubinow E, Ben Assa E, Rogowski O, Topilsky Y, Roth A (2013). Frequency and Correlates of Early Left Ventricular Thrombus Formation Following Anterior Wall Acute Myocardial Infarction Treated with Primary Percutaneous Coronary Intervention. *The American Journal of Cardiology*.

[10] Piccini JP, Stevens SR, Chang Y, Singer DE, Lokhnygina Y, Go AS (2013). Renal dysfunction as a predictor of stroke and systemic embolism in patients with nonvalvular atrial fibrillation: validation of the R(2)CHADS(2) index in the ROCKET AF (Rivaroxaban Once-daily, oral, direct factor Xa inhibition Compared with vitamin K antagonism for prevention of stroke and Embolism Trial in Atrial Fibrillation) and ATRIA (AnTicoagulation and Risk factors In Atrial fibrillation) study cohorts. *Circulation*.

[11] Gage BF, Waterman AD, Shannon W, Boechler M, Rich MW, Radford MJ (2001). Validation of Clinical Classification Schemes for Predicting Stroke: results from the National Registry of Atrial Fibrillation. *The Journal of the American Medical Association*.

[12] Lip GYH, Nieuwlaat R, Pisters R, Lane DA, Crijns HJGM (2010). Refining Clinical Risk Stratification for Predicting Stroke and Thromboembolism in Atrial Fibrillation Using a Novel Risk Factor-Based Approach: the euro heart survey on atrial fibrillation. *Chest*.

[13] Collins GS, Reitsma JB, Altman DG, Moons KGM (2015). Transparent reporting of a multivariable prediction model for individual prognosis or diagnosis (TRIPOD): the TRIPOD statement. *British Medical Journal*.

[14] Chaosuwannakit N, Makarawate P (2021). Left Ventricular Thrombi: Insights from Cardiac Magnetic Resonance Imaging. *Tomography*.

[15] Levine GN, McEvoy JW, Fang JC, Ibeh C, McCarthy CP, Misra A (2022). Management of Patients at Risk for and with Left Ventricular Thrombus: a Scientific Statement from the American Heart Association. *Circulation*.

[16] Miyasaka Y, Tsuji H, Tokunaga S, Nishiue T, Yamada K, Watanabe J (2000). Mild mitral regurgitation was associated with increased prevalence of thromboembolic events in patients with nonrheumatic atrial fibrillation. *International Journal of Cardiology*.

[17] Bosch J, Eikelboom JW, Connolly SJ, Bruns NC, Lanius V, Yuan F (2017). Rationale, Design and Baseline Characteristics of Participants in the Cardiovascular Outcomes for People Using Anticoagulation Strategies (COMPASS) Trial. *The Canadian Journal of Cardiology*.

[18] Khan F, Tritschler T, Kahn SR, Rodger MA (2021). Venous thromboembolism. *The Lancet*.

[19] Ming Ho K (2007). Forest and funnel plots illustrated the calibration of a prognostic model: a descriptive study. *Journal of Clinical Epidemiology*.

[20] Zhang Z, Gayle AA, Wang J, Zhang H, Cardinal-Fernández P (2017). Comparing baseline characteristics between groups: an introduction to the CBCgrps package. *Annals of Translational Medicine*.

[21] Cumpston M, Li T, Page MJ, Chandler J, Welch VA, Higgins JP (2019). Updated guidance for trusted systematic reviews: a new edition of the Cochrane Handbook for Systematic Reviews of Interventions. *The Cochrane Database of Systematic Reviews*.

[22] Arshad N, Bjøri E, Hindberg K, Isaksen T, Hansen J, Brækkan SK (2017). Recurrence and mortality after first venous thromboembolism in a large population‐based cohort. *Journal of Thrombosis and Haemostasis*.

[23] Áinle FN, Kevane B (2020). Which patients are at high risk of recurrent venous thromboembolism (deep vein thrombosis and pulmonary embolism)?. *Hematology*.

[24] Cushman M, Barnes GD, Creager MA, Diaz JA, Henke PK, Machlus KR (2020). Venous Thromboembolism Research Priorities: a Scientific Statement from the American Heart Association and the International Society on Thrombosis and Haemostasis. *Circulation*.

[25] Bechlioulis A, Lakkas L, Rammos A, Katsouras C, Michalis L, Naka K (2022). Venous Thromboembolism in Patients with Heart Failure. *Current Pharmaceutical Design*.

[26] Freudenberger RS, Hellkamp AS, Halperin JL, Poole J, Anderson J, Johnson G (2007). Risk of Thromboembolism in Heart Failure: an analysis from the Sudden Cardiac Death in Heart Failure Trial (SCD-HeFT). *Circulation*.

[27] Sosin MD, Bhatia G, Davis RC, Lip GYH (2003). Congestive Heart Failure and Virchow’s Triad: a Neglected Association. *Wiener Medizinische Wochenschrift*.

[28] Adelborg K, Szépligeti S, Sundbøll J, Horváth-Puhó E, Henderson VW, Ording A (2017). Risk of Stroke in Patients with Heart Failure: A Population-Based 30-Year Cohort Study. *Stroke*.

[29] Abdul-Rahim AH, Perez A, Fulton RL, Jhund PS, Latini R, Tognoni G (2015). Risk of Stroke in Chronic Heart Failure Patients without Atrial Fibrillation: Analysis of the Controlled Rosuvastatin in Multinational Trial Heart Failure (CORONA) and the Gruppo Italiano per lo Studio della Sopravvivenza nell’Insufficienza Cardiaca-Heart Failure (GISSI-HF) Trials. *Circulation*.

[30] Fanola CL, Norby FL, Shah AM, Chang PP, Lutsey PL, Rosamond WD (2020). Incident Heart Failure and Long-Term Risk for Venous Thromboembolism. *Journal of the American College of Cardiology*.

[31] Yilmaz H, Akkus C, Duran R, Diker S, Celik S, Duran C (2022). Neutrophil-to-lymphocyte and Platelet-to-lymphocyte Ratios in those with Pulmonary Embolism in the Course of Coronavirus Disease 2019. *Indian Journal of Critical Care Medicine*.

[32] Darzi AJ, Karam SG, Charide R, Etxeandia-Ikobaltzeta I, Cushman M, Gould MK (2020). Prognostic factors for VTE and bleeding in hospitalized medical patients: a systematic review and meta-analysis. *Blood*.

[33] Philip AM, George LJ, John KJ, George AA, Nayar J, Sahu KK (2021). A review of the presentation and outcome of left ventricular thrombus in coronavirus disease 2019 infection. *Journal of Clinical and Translational Research*.

[34] Sonaglioni A, Nicolosi GL, Lombardo M, Anzà C, Ambrosio G (2021). Prognostic Relevance of Left Ventricular Thrombus Motility: Assessment by Pulsed Wave Tissue Doppler Imaging. *Angiology*.

[35] Loh E, Sutton MS, Wun CC, Rouleau JL, Flaker GC, Gottlieb SS (1997). Ventricular dysfunction and the risk of stroke after myocardial infarction. *The New England Journal of Medicine*.

[36] Ibanez B, James S, Agewall S, Antunes MJ, Bucciarelli-Ducci C, Bueno H (2018). 2017 ESC Guidelines for the management of acute myocardial infarction in patients presenting with ST-segment elevation: The Task Force for the management of acute myocardial infarction in patients presenting with ST-segment elevation of the European Society of Cardiology (ESC). *European Heart Journal*.

[37] Lattuca B, Bouziri N, Kerneis M, Portal J, Zhou J, Hauguel-Moreau M (2020). Antithrombotic Therapy for Patients with Left Ventricular Mural Thrombus. *Journal of the American College of Cardiology*.

[38] Fleddermann AM, Hayes CH, Magalski A, Main ML (2019). Efficacy of Direct Acting Oral Anticoagulants in Treatment of Left Ventricular Thrombus. *The American Journal of Cardiology*.

[39] Giraud M, Catella J, Cognet L, Helfer H, Accassat S, Chapelle C (2021). Management of acute venous thromboembolism in patients taking antiplatelet therapy. *Thrombosis Research*.

[40] Parker WA, Storey RF (2021). Antithrombotic therapy for patients with chronic coronary syndromes. *Heart*.

[41] Brighton TA, Eikelboom JW, Mann K, Mister R, Gallus A, Ockelford P (2012). Low-Dose Aspirin for Preventing Recurrent Venous Thromboembolism. *New England Journal of Medicine*.

[42] Simes J, Becattini C, Agnelli G, Eikelboom JW, Kirby AC, Mister R (2014). Aspirin for the Prevention of Recurrent Venous Thromboembolism: the INSPIRE collaboration. *Circulation*.

[43] Cavallari I, Morrow DA, Creager MA, Olin J, Bhatt DL, Steg PG (2018). Frequency, Predictors, and Impact of Combined Antiplatelet Therapy on Venous Thromboembolism in Patients with Symptomatic Atherosclerosis. *Circulation*.

[44] Antiplatelet Trialists’ Collaboration (1994). Collaborative overview of randomised trials of antiplatelet therapy–III: Reduction in venous thrombosis and pulmonary embolism by antiplatelet prophylaxis among surgical and medical patients. *British Medical Journal*.

[45] Becattini C, Agnelli G, Schenone A, Eichinger S, Bucherini E, Silingardi M (2012). Aspirin for Preventing the Recurrence of Venous Thromboembolism. *New England Journal of Medicine*.

[46] Bastiany A, Grenier M, Matteau A, Mansour S, Daneault B, Potter BJ (2017). Prevention of Left Ventricular Thrombus Formation and Systemic Embolism after Anterior Myocardial Infarction: a Systematic Literature Review. *Canadian Journal of Cardiology*.

[47] Böhm M, Brueckmann M, Eikelboom JW, Ezekowitz M, Fräßdorf M, Hijazi Z (2020). Cardiovascular outcomes, bleeding risk, and achieved blood pressure in patients on long-term anticoagulation with the thrombin antagonist dabigatran or warfarin: data from the re-LY trial. *European Heart Journal*.

[48] Vidal-Petiot E, Ford I, Greenlaw N, Ferrari R, Fox KM, Tardif J (2016). Cardiovascular event rates and mortality according to achieved systolic and diastolic blood pressure in patients with stable coronary artery disease: an international cohort study. *The Lancet*.

[49] Sandhu RK, Ezekowitz J, Andersson U, Alexander JH, Granger CB, Halvorsen S (2016). The ‘obesity paradox’ in atrial fibrillation: observations from the ARISTOTLE (Apixaban for Reduction in Stroke and other Thromboembolic Events in Atrial Fibrillation) trial. *European Heart Journal*.

[50] Balla SR, Cyr DD, Lokhnygina Y, Becker RC, Berkowitz SD, Breithardt G (2017). Relation of Risk of Stroke in Patients with Atrial Fibrillation to Body Mass Index (from Patients Treated with Rivaroxaban and Warfarin in the Rivaroxaban once Daily Oral Direct Factor Xa Inhibition Compared with Vitamin K Antagonism for Prevention of Stroke and Embolism Trial in Atrial Fibrillation Trial). *The American Journal of Cardiology*.

[51] Boriani G, Ruff CT, Kuder JF, Shi M, Lanz HJ, Rutman H (2019). Relationship between body mass index and outcomes in patients with atrial fibrillation treated with edoxaban or warfarin in the ENGAGE AF-TIMI 48 trial. *European Heart Journal*.

