# Supplementary material for: Predicting Thromboembolism in Hospitalized Patients with Ventricular Thrombus
Source: Rev Cardiovasc Med. 2022 Nov 30;23(12):390. doi: 10.31083/j.rcm2312390 (PMC11270478; doi:10.31083/j.rcm2312390)
Supplement: Supplementary file 1 [file 2153-8174-23-12-390-s1.zip › 2153-8174-23-12-390-s1/Supplementary File.docx]

**Predicting Thromboembolism in Hospitalized Patients with Ventricular Mural Thrombus**

[Figure and Table Legends 2](#_Toc116114427)

[Supplementary Figure 3](#_Toc116114428)

[Supplementary Table 10](#_Toc116114429)

Figure and Table Legends

**Supplementary Fig. 1. Flow diagram to show the inclusion and exclusion criteria.** 498 patients were found with ventricular thrombus and 338 patients were included in the analysis. †Others included 5 patients who had a suspected diagnosis of thrombophilia at discharge. N, numbers of patients.

**Supplementary Fig. 2. Changes in 46 factors with the penalty parameter (Lambda).**

**Supplementary Fig. 3. ROC curves of Model 1 for predicting the risk of thromboembolism.** (A) Training set. (B) Validation set. ROC, receiver operating characteristic; AUC, area under the ROC curve.

**Supplementary Fig. 4. ROC curves of Model 1 and Model 2 for predicting the risk of thromboembolism.** (A) Training set. (B) Validation set. ROC, receiver operating characteristic.

**Supplementary Fig. 5. Calibration plots for predicting the risk of thromboembolism in the validation set of Model 1.** (A) Training set. (B) Validation set. X-axis, bootstrap-predicted probability; Y-axis, actual probability.

**Supplementary Fig. 6. Calibration plots for predicting the risk of thromboembolism in the validation set of Model 2.** (A) Training set. (B) Validation set. X-axis, bootstrap-predicted probability; Y-axis, actual probability.

**Supplementary Fig. 7. DCA of the novel nomogram for predicting the risk of thromboembolism in Model 1 and Model 2.** (A) Training set. (B) Validation set. X-axis, bootstrap-predicted probability; Y-axis, actual probability; DCA, decision curve analysis.

**Supplementary Table 1. Main factors at discharge with the reference of baseline value in the training group and validation group**

Variables are presented as n (%).

Abbreviations: N, numbers of patients; FDP, fibrin degradation products; NT-proBNP, N-Terminal pro-brain natriuretic peptide.

Supplementary Figure

**Supplementary Fig. 1. Flow diagram to show the inclusion and exclusion criteria.** 498 patients were found with ventricular mural thrombus and 338 patients were included in the analysis. †Others included 5 patients who had a suspected diagnosis of thrombophilia at discharge. N, numbers of patients.

**Supplementary Fig. 2. Changes in 46 factors with the penalty parameter (Lambda).**

(A)

(B)

**Supplementary Fig. 3. ROC curves of Model 1 for predicting the risk of thromboembolism.** (A) Training set. (B) Validation set. ROC, receiver operating characteristic; AUC, area under the ROC curve.

(B)

(A)

**Supplementary Fig. 4. ROC curves of Model 1 and Model 2 for predicting the risk of thromboembolism.** (A) Training set. (B) Validation set. ROC, receiver operating characteristic.

(A)

(B)

**Supplementary Fig. 5. Calibration plots for predicting the risk of thromboembolism in the validation set of Model 1.** (A) Training set. (B) Validation set. X-axis, bootstrap-predicted probability; Y-axis, actual probability.

(A)

(B)

**Supplementary Fig. 6. Calibration plots for predicting the risk of thromboembolism in the validation set of Model 2.** (A) Training set. (B) Validation set. X-axis, bootstrap-predicted probability; Y-axis, actual probability.

(B)

(A)

**Supplementary Fig. 7.** **DCA of the novel nomogram for predicting the risk of thromboembolism in Model 1 and Model 2.** (A) Training set. (B) Validation set. X-axis, bootstrap-predicted probability; Y-axis, actual probability; DCA, decision curve analysis.

Supplementary Table

Supplementary Table 1. Main factors at discharge with the reference of baseline value in the training group and validation group

|  | **Total**  **(N=338)** | **Training group**  **(N=238)** | **Validation group**  **(N=100)** | **P value** |
| --- | --- | --- | --- | --- |
| D-dimer, ug/ml |  |  |  | 0.937 |
| -1 ~ +1fold | 201 (59.5) | 143 (60.1) | 58 (58) |  |
| +1fold~ | 42 (12.4) | 29 (12.2) | 13 (13) |  |
| ~-1fold | 95 (28.1) | 66 (27.7) | 29 (29) |  |
| FDP, ug/ml |  |  |  | 0.392 |
| -1 ~ +1fold | 277 (82) | 191 (80.3) | 86 (86) |  |
| +1fold~ | 26 (7.7) | 21 (8.8) | 5 (5) |  |
| ~-1fold | 35 (10.4) | 26 (10.9) | 9 (9) |  |
| NT-proBNP, pg/ml |  |  |  | 0.140 |
| -1 ~ +1fold | 177 (52.4) | 122 (51.3) | 55 (55) |  |
| +1fold~ | 38 (11.2) | 32 (13.4) | 6 (6) |  |
| ~-1fold | 123 (36.4) | 84 (35.3) | 39 (39) |  |

Variables are presented as n (%).

**Abbreviations:** N, numbers of patients; FDP, fibrin degradation products; NT-proBNP, N-Terminal pro-brain natriuretic peptide.
