# Supplementary figures and images for: Predicting Thromboembolism in Hospitalized Patients with Ventricular Thrombus
Source: Rev Cardiovasc Med. 2022 Nov 30;23(12):390. doi: 10.31083/j.rcm2312390 (PMC11270478; doi:10.31083/j.rcm2312390)

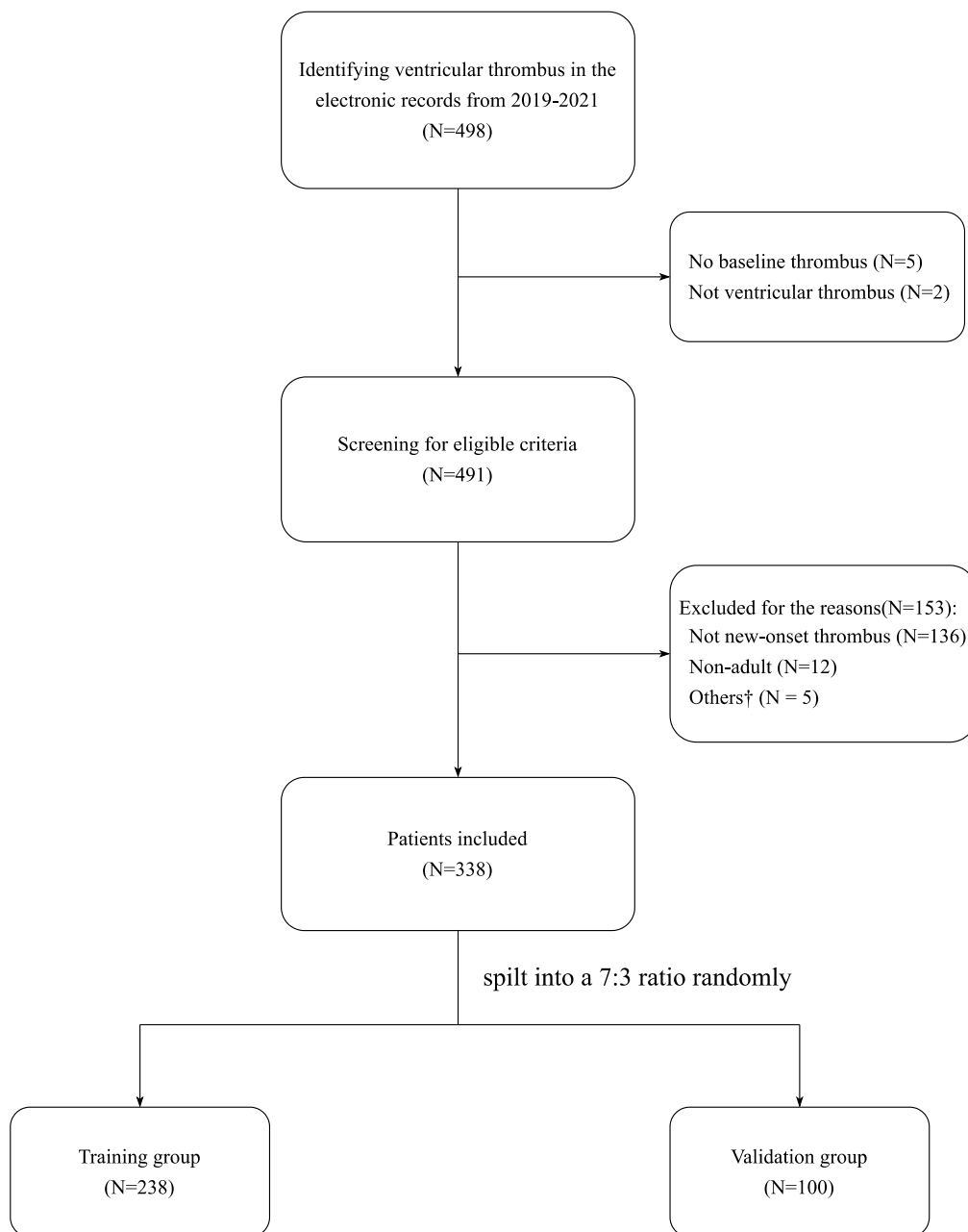

Supplement: Supplementary file 1 [file 2153-8174-23-12-390-s1.zip › 2153-8174-23-12-390-s1/Figure S1.pdf]
